# Workplace cluster of Bell’s palsy in Lima, Peru

**DOI:** 10.1186/1756-0500-7-289

**Published:** 2014-05-09

**Authors:** Erik J Reaves, Mariana Ramos, Daniel G Bausch

**Affiliations:** 1 U.S. Naval Medical Research Unit No. 6, Department of Emerging Infections, Unit 3230, Box 337, DPO, AA 34031 Lima, Peru; 2 Tulane School of Public Health and Tropical Medicine, New Orleans, LA, USA

**Keywords:** Bell’s palsy, Facial paralysis, Workplace cluster, Outbreak

## Abstract

**Background:**

We report on a workplace cluster of Bell’s palsy that occurred within a four-month period in 2011 among employees of a three-story office building in Lima, Peru and our investigation to determine the etiology and associated risk factors.

**Findings:**

An outbreak investigation was conducted to identify possible common infectious or environmental exposures and included patient interviews, reviews of medical records, an epidemiologic survey, serological analysis for IgM and IgG antibodies to putative Bell’s palsy-inducing pathogens, and an environmental exposure assessment of the office building. Three cases of Bell’s palsy were reported among 65 at-risk employees, attack rate 4.6%. Although two patients had underlying risk factors, there was no clear association or common identifiable risk factor among all cases. Serologic analysis showed no evidence of recent infections, and air and water sample measures of all known chemical or neurotoxins were below maximum allowable concentrations for exposure.

**Conclusions:**

An infection spread among workplace employees could not be excluded as a potential cause of this cluster; however, it was unlikely a pathogen commonly associated with individual cases of Bell’s palsy. Although a specific etiology was not identified among all cases, we believe this methodology will aid future outbreak investigations of Bell’s palsy and a better understanding of its etiology. While environmental assessments may be useful in their ability to ascertain the cause of clusters of Bell’s palsy, future investigations should prioritize focus on common infectious etiology.

## Findings

### Introduction

Bell’s palsy, an acute facial paralysis affecting the 7^th^ cranial nerve, comprises up to half of all peripheral facial palsies. Paralysis is typically unilateral. The incidence in the general population ranges from 15-30 cases per 100,000 person-years and is roughly equal among men and women [1]. Peak incidence occurs in the fifth decade of life and the syndrome is more common in patients with diabetes and in pregnant women [2]. There have been reports of increased incidence in cooler, winter months, although precise relationships with climate and season remain to be elucidated [3,4]. Other conditions known to produce facial nerve palsy include structural lesions of the ear or parotid gland, Guillain-Barré syndrome, Ramsay Hunt syndrome, otitis media, sarcoidosis, and influenza vaccination, although these conditions typically have additional distinguishing features from Bell’s palsy [2].

The cause of Bell’s palsy is unknown. Infectious etiologies have been hypothesized, including infection or reactivation of neurotropic herpes viruses, namely herpes simplex virus type 1 (HSV-1) and varicella-zoster virus (VZV), HIV/AIDS, and Lyme disease (*Borrelia burgdorferi*), resulting in peripheral neuropathy [5-7]. Antecedent respiratory infections have also been postulated to be a cause [8]. Although an analysis using the Vaccine Adverse Event Reporting System in the U.S. found an increase of Bell’s palsy cases in the 3-month period following parenteral inactivated influenza vaccination [9], no evidence of increased risk was found in a subsequent self-controlled case-series study using the General Practice Research Database [10]. Bell’s palsy associated with hypothyroidism has also been reported [11].

Up to 85 percent of patients with Bell’s palsy recover spontaneously without treatment [12]. For patients who present within 72 hours of onset of symptoms, a tapering, short-course of oral corticosteroids to reduce facial nerve inflammation has been shown to increase the return of facial function [2,13]. Treatment with acyclovir or valacyclovir aimed at possible HSV-1 infection is of uncertain benefit.

Rarely, temporal or geographic clusters of Bell’s palsy have been reported, suggesting a possible common infectious or toxic exposure, although a definitive etiology has yet to be identified [4,8,14]. We report on a small workplace cluster of cases of Bell’s palsy in urban Lima, Peru, and our investigation to attempt to identify a cause and characterize risk factors among workplace employees.

### The outbreak and investigation

Between April 20^th^ and August 23^rd^, 2011, three cases of Bell’s palsy were reported among 65 employees (attack rate 4.6%) at a three-story office work place in Lima, Peru. Support was requested from administrative staff that occupied the office building in Lima on September 1^st^, 2011. In response, we conducted an investigation to confirm the existence of an outbreak, identify risk factors, and explore possible infectious and environmental etiologies. Activities of the outbreak investigation team included patient interviews, reviews of medical records, an epidemiologic survey of workplace employees, and laboratory analysis for infections that could predispose to Bell’s palsy. Survey responses and serum samples were obtained by voluntary consent and all names and survey and test results were kept strictly confidential. In addition, an environmental exposure assessment was arranged by the work place administration.

The majority of the employees at the work site are Peruvian. All three patients worked in the same office building but at different workstations. Patients 1 and 3 worked on different floors while Patient 2 worked primarily outside on the building’s exterior grounds. None of the patients lived together or reported common exposures outside the work place. By their self-report, the patients were casual work place acquaintances but did not have particularly close contact or interactions either during or after work hours. The patients were medically evaluated and treated by their primary healthcare practitioner. The clinical features were consistent with what has been previously described for Bell’s palsy [1]. Clinical and demographic characteristics of each case are presented in Table 1.

**Table 1:** Characteristics of three patients with Bell’s palsy

**Characteristic**	**Patient 1**	**Patient 2**	**Patient 3**
***Date of Onset***	04/20/2011	06/14/2011	08/23/2011
***Age***	48	46	60
***Gender***	Female	Male	Female
***Nationality***	Peruvian	Peruvian	Peruvian
***Affected Side***	Right	Left	Right
***Severity***	Mild	Mild	Moderate
***Duration***	4 weeks	4 weeks	5 weeks
***Medical Treatment***	Ac, B, S, PT	Ac, NSAIDs, PT	NSAIDs, S, V
***Resolution***	Complete	Complete	Complete
***Recurrence***	No	No	No
***Prior contact with person with BP***	No	No	No
***Past Medical History***	Recurrent otitis media (last episode February 2 months prior to BP), Adult chickenpox in 2000	Childhood chickenpox	URI 2 months prior to BP, Hypothyroidism, Non-insulin-dependent diabetes mellitus, Childhood chickenpox
***Routine Medications***	None	None	levothyroxine, metformin, isotretinoin, flutamide, calcium, MVI
***Preceding Vaccinations***	None	None	Influenza vaccine 05/31/2011
***Tobacco use***	No	No	No
***Preceding Travel***	Southeastern U.S. 7 months prior to BP	None	Western Europe one week prior to BP

*Abbreviations*: *Ac* acyclovir, *B* vitamin B complex, *BP* Bell’s Palsy, *NSAIDs* non-steroidal anti-inflammatory drugs, *S* oral corticosteroids, *V* valacyclovir, *PT* physiotherapy, *URI* Upper respiratory illness.

To evaluate for possible infectious etiologies, serum samples were tested by enzyme-linked immunosorbent assay and compliment fixation for IgM and IgG antibodies to Coxsackie B, cytomegalovirus, Epstein-Barr virus, HSV-1, influenza A and B, paramyxovirus, and varicella zoster viruses, *Mycoplasma pneumoniae*, and *Haemophilus influenzae*. Two serum samples were obtained from each patient, on September 1^st^ and October 25^th^, 2011, which was 4-8 months after the onset of symptoms for the three patients. Only Patient 3 still had facial paralysis at the time of testing and of our investigation. Testing was performed at a private laboratory in Lima using commercially available kits (Diagnostic Automation, Inc.; Quest Diagnostics; Roche Cobas; Vircell).

All three patients had IgG antibody titers indicative of previous exposure or vaccination to all the tested pathogens, with the exception of Patient 3, who was IgG negative for HSV-1. All IgM tests were negative. Although ticks carrying *B. burgdorferi* variants have been reported in some areas of South America with rare reports of associated human Lyme disease, no cases have ever been reported in Peru and therefore testing for Lyme disease was not readily available or conducted [15]. We were not able to test the patients for HIV infection, but the prevalence of HIV/AIDS in Peru is low (<1%) and none of the patients are in the identified high-risk age group (25-34 years) for HIV infection in the country or reported risk factors for newly acquired HIV infection (Peru Ministry of Health and USAID statistics).

To evaluate for a possible common environmental exposure, a building air and water quality assessment was performed by a contracted, commercial industrial hygiene consult service with expertise in indoor pollution. The building had a shared, re-circulated air-handling system between its three floors. The ventilation system and work centers were inspected to measure for known chemical and neurotoxins to detect possible sources of bioaerosoles and hazardous airborne contaminants. Point sources for potable water were sampled and levels of various metals, bacteria, and volatile organic compounds were measured. Samples were collected and analyzed according to guidelines established by the National Institute of Occupational Safety and Health (NIOSH). All environmental samples measured below the maximum allowable level set by local and NIOSH standards.

This study was approved by the U.S. Naval Medical Research Unit No. 6 Institutional Review Board in compliance with all applicable U.S. Federal regulations governing the protection human subjects. Survey responses and serology samples were voluntary and analyzed de-identified. The three patients with Bell’s palsy provided written consent for the investigators of this outbreak to publish non-personal identifiable information related to the clinical aspects of their conditions.

## Discussion

We investigated the hypotheses that the cause of this cluster was either infectious or a shared environmental exposure in the workplace. From our review of the literature, this is the fourth reported cluster of cases of Bell’s palsy [4,8,14]. As with the other three reports, we were unable to identify a specific etiology for the cases collectively or individually. Although we were not able to perform a case-control study to compare relative risks of Bell’s palsy, the broadly positive IgG serological results of the three patients with Bell’s palsy are no doubt typical of the general population, since almost everyone is exposed at some point to these common pathogens. Furthermore, the negative IgM serology results suggest that no recent infection with these pathogens occurred, although the IgM response in reinfection or reactivation may be blunted [16].

HSV-1 has been suggested as a possible nonepidemic cause because of elevated antibody titers in patients affected with Bell’s palsy; however, viral DNA is inconsistently isolated during acute infection and has been present in healthy study controls [6]. In this analysis, Patient 3 did not have IgM or IgG antibody titers to HSV-1, excluding primary infection or reactivation as a cause of this cluster and further questioning its role in the pathogenesis of clinical Bell’s palsy. VZV reactivation without rash i.e. *zoster sine herpete* may have been the etiology given the previous history of varicella infection in each patient; however, whether this occurred by chance or constituted some common predisposing pathway remains unclear. In addition, since blood samples for laboratory testing were taken months after symptom onset, and after resolution of symptoms in two of the three patients, we cannot completely exclude that acute infection with one of the tested pathogens occurred, either in the absence of detectable antibody response or with subsequent reversion of IgM titer back to negative before serum collection for testing occurred.

Although we were unable to test for Lyme disease, we believe it is an unlikely explanation for these cases. While Patient 1 traveled to the southeastern United States where Lyme disease endemicity is highest, the travel occurred 7 months prior to disease onset. Patient 3 had recently traveled to an area where Lyme disease is endemic, and during the typical season of transmission, but did not report significant outdoor activities or exposure to ticks during the trip and had no symptoms consistent with *B. burgdorferi* infection, such as erythema migrans or arthritis. Although we do not consider HIV infection as a likely etiology of these cases, it cannot be completely excluded since we were unable to arrange testing for this virus.

There was no obvious environmental toxic etiology of the cases. Although the air in Lima is among the most polluted in Latin America, surpassing the international standard established by the American Agency of Environmental Protection by 122.1 percent (Peru National Statistics and Information Institute, 2006), the air and water quality assessment did not reveal exposure risks inside the building. If observed cases of Bell’s palsy resulted from an environmental exposure, there likely would have been other individuals with clinical or subclinical evidence of paralysis; however, no other office staff had signs or symptoms of facial nerve dysfunction or reported chemical or toxic environmental exposure in the workplace. Furthermore, Bell’s palsy is not recognized as an outcome of neurotoxic chemical exposure, nor are cranial nerves often affected by such exposures [14]. Concentrations of measured contaminants in air and water quality samples were all below detection or applicable exposure standards and did not reveal a pattern consistent with disease risk. Nonetheless, subclinical facial nerve dysfunction that could be present with chemical neurotoxin exposure may have occurred among clinically healthy employees but was not evident in our investigation. Investigations of possible toxins in two other outbreaks of Bell’s palsy also failed to reveal any obvious environmental cause [8,14].

A number of possible predisposing conditions, including diabetes and hypothyroidism, and triggering events, including recent (ie. with ~2 months) influenza vaccination and otitis media and upper respiratory infection of unknown etiologies, were noted for the three patients. However, these conditions and events were not uniform across all three cases, and thus cannot be invoked as common factors for the cluster, although they may be implicated for an individual case. It should be noted that all of these conditions and events are also common in the general population in Lima.

In the absence of a shared cause for Bell’s palsy, it is possible that the clustering of these three cases occurred by chance, although this would seem unlikely when we consider that, according to the records and memory of people at the work place, no case of Bell’s palsy had ever been reported there before experiencing an attack rate of 4.6% (4,615 per 100,000 person-years) over a four-month period in 2011. By comparison, in the district where the office building is located and the Bell’s palsy cluster occurred, with an estimated population of 85,284 from the 2011 census, the Peru Ministry of Health recorded five cases of Bell’s palsy in 2010 and four in 2011, excluding the cases reported here. This corresponds to attack rates of less than 0.0059%. Bell’s palsy is not a reportable disease in Peru so these numbers may be under-estimates. Interestingly, the cluster in Lima occurred during the fall and winter months, consistent with previous observations of increased incidence in winter months in the southern hemisphere [4].

## Conclusions

Although we were unable to identify a specific etiology for this outbreak of Bell’s palsy, we believe that it is nevertheless important that these “negative data” appear in the scientific literature to aid in the investigation of possible future cases and clusters, hopefully eventually leading to identification of a specific etiology and ultimately control and prevention measures. We believe that the causes of Bell’s palsy are probably multifactorial, involving infectious, environmental, and/or emotional triggers super-imposed on persons predisposed through underlying microvascular or neurologic impairment due to chronic conditions such as diabetes mellitus.

To our knowledge only two other investigations of an outbreak of Bell’s palsy included an assessment to characterize the role of environmental exposures as a possible etiology [8,14]. While serving an important public health purpose to belay concerns of fearful employees, our investigation results further support the conclusion that there is limited additional benefit in conducting an environmental exposure assessment to ascertain causality in outbreaks of Bell’s palsy. Future investigations might instead focus on common infectious etiology.

## Competing interests

The authors declare that they have no competing interests.

## Authors’ contributions

EJR and MR conducted the outbreak investigation with guidance on study design and data acquisition, analysis, and interpretation from DGB. All three authors contributed to the writing of this manuscript and give final approval of this version to be published.

## Copyright statement

Some authors are military service members of the U.S. Government. This work was prepared as part of their official duties. Title 17 U.S.C. §105 provides that ‘Copyright protection under this title is not available for any work of the United States Government.’ Title 17 U.S.C. §101 defines a U.S. Government work as a work prepared by a military service member or employee of the U.S. Government as part of that person’s official duties.
